# 肺癌PD1/PD-L1免疫检查点治疗疗效预测标志物第18届世界肺癌大会相关研究综述

**DOI:** 10.3779/j.issn.1009-3419.2018.09.09

**Published:** 2018-09-20

**Authors:** 冠璇 陈, 现让 宋

**Affiliations:** 1 250117 济南，山东大学附属山东省肿瘤医院ICU ICU, Affliated to Shandong University, Ji'nan 250117, China; 2 250117 济南，山东大学附属山东省肿瘤医院 基础实验室 Basic Laboratory, Shandong Tumor Hospital, Affliated to Shandong University, Ji'nan 250117, China

**Keywords:** 肺肿瘤, PD1/PD-L1, 标志物, 世界肺癌大会, Lung neoplasms, PD1/PD-L1, Biomarkers, World Conference on Lung Cancer

## Abstract

程序化死亡受体-1（programmed cell death-1, PD-1）/程序化死亡受体的配体（programmed cell death-ligand 1, PD-L1）免疫检查点抑制剂治疗非小细胞肺癌（non-small cell lung cancer, NSCLC）是近年来最引人注目的进展之一，但目前尚无明确的疗效预测标志物，研究者期望找到明确的标志物来筛选免疫治疗有效的患者，最大程度地让患者获益。第18届世界肺癌大会有关PD-1/PD-L1免疫治疗疗效预测的标志物的研究成为热点。本综述整理分析本次大会上关于肺癌免疫治疗标志物的内容，归纳为以下6个方面：PD-L1表达、肿瘤突变负荷及基因突变修复能力、肿瘤驱动基因突变、免疫效应标志物、血细胞计数、综合分析模型，以帮助临床医生选择合适的生物标志物来筛选能够获益的患者和对治疗疗效进行有效评估。

## Abstract (en)

Programmed cell death-1 (PD-1)/programmed cell death-ligand 1 (PD-L1) checkpoint blockades have dramatically changed the treatment of non-small cell lung cancer (NSCLC). But we still have no definite biomarkers that may predict the efficacy of treatment by PD-1/PD-L1 inhibitors. In the 18^th^ World Conference on Lung Cancer, the biomarkers that may predict the efficacy of treatment by PD-1/PD-L1 inhibitors in patients with lung cancer has been a popular topic, and it has huge potential in the future. In order to enable more patients to get more benefits from treatment, researchers are looking forward to finding the optimum biomarkers. By organizing and summarizing the information about the biomarkers predicting PD-1/PD-L1 in patients with lung cancer, this review mainly focused on the following six aspects to introduce: expression of PD-L1; tumor mutational burden and the ability of mutation repair, malignant tumor driver mutation, biomarker of immunological effect, blood cell account, comprehensive analysis model. We are hoping to help doctors to find the best biomarker, then much more lung cancer patients could obtain antitumor effects in PD-1/PD-L1 inhibitors treatment.

在肿瘤细胞和免疫效应细胞的相互作用中，肿瘤细胞表面的程序化死亡受体的配体（programmed cell death-ligand 1, PD-L1）与免疫效应细胞（T淋巴细胞）表面的程序化死亡受体-1（programmed cell death-1, PD-1）的结合，以及肿瘤细胞表面的CD80/CD86分化抗原与T淋巴细胞表面的CTLA-4的结合，传递免疫抑制信号，是肿瘤细胞逃避淋巴细胞杀伤的重要机制。针对这两个抑制信号途径的免疫靶向治疗是近年来最令人瞩目的进展之一。

2017年10月在日本横滨召开的第18届世界肺癌大会上，肿瘤免疫治疗是一个重要的主题，不仅有多项有关肺癌免疫治疗的临床试验被与会者关注，有关免疫治疗疗效预测的标志物的研究也是热点之一。研究者期望如同肺癌靶向治疗一样找到明确的标志物来筛选免疫治疗有效的患者，最大程度地让患者获益，避免不必要的昂贵且无效的治疗。本文通过整理分析本次大会上关于肺癌免疫治疗标志物的内容，以帮助临床医生选择合适的生物标志物来筛选能够获益的患者和对治疗疗效进行有效评估。

## 1 PD-L1表达

Nivolumab、pembrolizumab和atezolizumab的Checkmate 026、Keynote-010和OAK研究均表明PD-1/PD-L1抑制剂在NSCLC患者中的疗效与肿瘤细胞PD-L1的表达水平相关。食品药品监督管理局（Food and Drug Administration, FDA）已经批准了单药Pembrolizumab用于PD-L1表达水平≥50%的非小细胞肺癌（non-small cell lung cancer, NSCLC）患者的一线治疗。但是PD-L1作为PD-1/PD-L1抑制剂的疗效预测指标尚存较大争议，Checkmate 017研究发现PD-L1表达水平与PD-1抑制剂在肺鳞癌中的疗效无关。部分PD-L1阴性患者也可以从PD-1/PD-L1抑制剂治疗中获益。而且不同平台间检测PD-L1表达水平的一致性不佳，尚没有得到普遍认可的检测方法，PD-L1的Cut-off值如何确定也争议不断，PD-L1表达到什么程度可以应用免疫治疗目前并不明确。

在本次大会上，有几项研究分析了与PD-L1表达相关的临床病理因素。例如有研究表明PD-L1在肺鳞癌患者中表达率高于腺癌，与其他因素如性别、年龄、是否吸烟、病理类型、肿瘤分期均没有统计学相关性^[1-3]^。Shi等^[4]^统计分析了来自34项临床试验的9, 934例患者，PD-L1高水平表达与表皮生长因子受体（epidermal growth factor receptor, EGFR）野生型（OR=0.68, 95%CI: 0.48-0.96, *P*=0.03）、非腺癌（OR=0.68, 95%CI: 0.47-0.98, *P*=0.04）、*KRAS*突变（OR=1.27, 95%CI: 1.02-1.58, *P*=0.03）明显相关。大部分研究认为PD-L1高水平表达与EGFR野生型及*KRAS*突变是相关的，但几乎所有相关研究结果都表明PD-L1表达与ALK不存在相关性^[5, 6]^。

除以上因素外，化疗也有可能影响PD-L1表达，一项研究观察了术前明确病理并接受新辅助化疗的26例患者化疗前后PD-L1的表达水平。以5%、10%、20%为阳性界定标准时，化疗前PD-L1阳性率分别为65.4%（17/26）、53.8%（14/26）、42.3%（11/26），而化疗后的阳性率为92.3%（24/26）、80.8%（21/26）、69.2%（18/26），化疗后PD-L1表达明显上调（*P*=0.003, *P*=0.016, *P*=0.016）。当以30%、50%为界定阳性标准时，化疗前后的PD-L1表达没有统计学意义。化疗前后PD-1、PD-L1、PD-L2的表达水平的改变与病理类型，化疗结束至手术的时间间隔、性别、吸烟均不存在相关性。该研究的局限性在于没有分析同一患者化疗前后PD-L1表达水平的变化，而且化疗造成的表达改变是否可以影响疗效，尚在研究中^[2]^。值得注意的是，肿瘤组织的异质性可能是导致活检组织PD-L1表达水平低于切除组织的原因^[6]^，而本研究术前为活检组织，术后为切除组织，所以其现实意义，有待进一步证明。

PD-L1表达的相关因素，有一项研究结果与其他研究不尽相同，该选择接受手术治疗的508例患者，以1%为临界点判定PD-L1阳性，结果阳性者为188例（37.0%）。多因素*Logistic*回归分析PD-L1阳性表达与较晚分期、血管密度、高水平C反应蛋白（C-reactive protein, CRP）相关。吸烟指数与高水平CRP与PD-L1表达明显相关^[5]^。这可能与不同研究选择的临界点不同有一定关系。关于血管密度，有研究证明前期使用抗血管生成药物的患者，再接受免疫抑制剂效果不佳，无进展生存期（progression-free survival, PFS）（*P*=0.044, 4）、总生存期（overall survival, OS）（*P*=0.074, 1）显著缩短^[7]^。

除了检测肿瘤组织PD-L1的表达水平外，循环肿瘤细胞（circulating tumor cells, CTCs）也可用来检测PD-L1表达水平。Tanizaki等^[8]^报道，晚期NSCLC患者nivolumab治疗前收集外周血并分离CTCs，该研究结果显示，CTCs的PD-L1表达不能预测药物疗效，但是，PFS < 6个月组CTCs的PD-L1表达水平明显要高于PFS > 6个月组。所以，CTCs的PD-L1表达水平虽然不能预测nivolumab疗效，但是其对预后的评估作用不可忽视^[9]^。

另外有检测可溶性抗原的研究。一项入组22例患者，使用PD-L1/PD-1抑制剂的研究^[10]^结果表明sPD-L1（soluble PD-L1）与肿瘤组织中PD-L1表达水平无相关性，虽然OS及PFS跟sPD-L1没有相关性，但是sPD-L1低水平组的OS要高，特别是腺癌患者。Kang等^[3]^检测了2014年4月-2017年1月的164例肺癌患者治疗前后血浆中的sPD-L1水平，同时测定了73例健康人的sPD-L1水平作为对照组。结果发现，肿瘤患者血浆中sPD-L1水平高于肺部良性疾病的患者。sPD-L1低表达组的OS高于高表达组（28.0个月*vs* 12.0个月，*P* < 0.001）两组的PFS没有差异。化疗有效患者的sPD-L1水平明显下降，而病情进展患者的sPD-L1水平明显升高。*EGFR*突变患者的sPD-L1明显高于野生型患者。sPD-L1水平与年龄、性别、是否吸烟、病理类型、肿瘤分期没有相关性。

综合上述研究的结果分析，肿瘤细胞PD-L1的表达水平与很多因素相关，所以，并不是全部高表达PD-L1的患者均能在免疫治疗中获益。在正常情况下，激活PD-1/PD-L1信号通路可以诱导、维持免疫耐受，阻止过度的炎症反应对机体的伤害，并在自身免疫性疾病的发生具有积极作用。当抗原清除，免疫应答完成后，T细胞的PD-1表达减少。在肿瘤组织中，PD-L1是由T细胞分泌的γ-干扰素（interferon-γ, IFN-γ）诱导表达的，PD-L1在肿瘤细胞表面的表达量增加意味着免疫细胞抗肿瘤活性的进行。PD-1持续性在T细胞表达会诱导T细胞的衰竭，所以，笔者认为PD-1/PD-L1的表达本身是动态变化的，在某一时间点的检测值，可能并不能完全反映整体的实际情况，可能需要结合其他反映免疫反应的因素进行分析。

机体抗肿瘤效应的发挥不仅依赖于PD-L1介导的抑制性信号的解除，还依赖于细胞毒性T细胞。因此同时检测组织PD-L1及CD8^+^细胞的状态可能更有预测价值。Wu等^[1]^根据肿瘤微环境的免疫状态将肺癌患者分为4型，即type Ⅰ（PD-L1^+^ CD8^+^）、type Ⅱ（PD-L1^-^ CD8^-^）、type Ⅲ PD-L1^+^ CD8^-^）和type Ⅳ（PD-L1^-^ CD8^+^），共入组58例肺癌早期患者，大部分为腺癌Ⅰ期患者。PD-L1表达阳性率为74.14%（43/58），低表达为60.34%（35/58），中度表达29.31%（17/58），高表达1.72%（1/58）。Ⅰ型72.41%（42/58），Ⅱ型6.90%（4/58），Ⅲ型1.72%（1/58），Ⅳ型18.97%（11/58）。PD-L1^+^ CD8^+^最多，PD-L1^+^ CD8^-^占比最少。该研究发现，CD8^+^ T细胞浸润情况跟年龄、性别、分期及病理类型均没有相关性。所以，该项研究者认为，应用免疫抑制剂治疗前，检测组织PD-L1及CD8^+^细胞的状态可以提高有效率。但是，壁报中并未展示不同分型患者治疗获益的相关数据，该研究的进一步数据，非常值得期待。

相似的研究来自Wang等^[11]^，检测了168例肺神经内分泌肿瘤组织PD-L1及CD8^+^ TILs的状态。肿瘤细胞PD-L1表达水平≥5%或TILs PD-L1表达水平 > 1%界定为PD-L1阳性。72例患者的肿瘤细胞或TILs表面均有PD-L1表达，占总数的42.9%。CD8^+^ TILs与PD-L1表达有显著相关性。PD-L1与OS及PFS没有相关性。肿瘤间质CD8^+^ TIL越多OS及PFS越长，并且是良好预后的独立相关因素。

黑色素瘤免疫检查点抑制剂治疗开始较肺癌更早，预测其疗效标志物的研究更加深入。一项研究^[12]^认为，CD8^+^细胞的总数并不是黑色素瘤预后的关键，起决定作用的是CD69^+^ CD103^+^肿瘤驻留CD8^+^ T细胞数量，在这些肿瘤驻留CD8^+^ T表面PD1的表达更多，同时这类细胞在使用免疫抑制剂治疗早期会显著增殖。研究者认为，肿瘤驻留CD8^+^ T有可能会成为预测免疫抑制剂疗效的标志物。

## 2 肿瘤突变负荷及基因突变修复能力

肿瘤突变负荷（tumor mutational burden, TMB）是指每百万碱基中被检测出的体细胞基因编码错误、碱基替换、基因插入或缺失错误的总数。理论上TMB越高，新的肿瘤相关抗原的产生就越多，就越有可能刺激产生免疫应答，配合抑制性免疫信号的解除，治疗效果会越好^[13]^。错配修复基因（mis-match repair, MMR）编码的蛋白与基因的错配修复有关。MMR功能缺陷造成微卫星不稳定（microsatelliteinstability, MSI）和体细胞基因变异得不到修复，TMB也就会高。MSI本身就是一种带有微卫星变化的基因超突变状态。通常而言，绝大部分MSI-H的样本，TMB也较高，但反之则不一定成立。鉴于MMR、MSI和TMB三者之间的相互关系，三者均有作为免疫检查点抑制剂治疗标志物的理论可行性。

TMB检测的方法是基因组分析，方法包括全基因组测序、全外显子测序和选择性基因测序，有研究认为不同大小的测序组合会影响TMB准确度。TMB的cut-off值以及不同瘤种间是否存在差异还没有统一的标准，目前普遍认同 < 6 mutations/Mb定义为TMB低，≥20 mutations/Mb定义为TMB高。

基因分析发现，在肺癌中携带下述基因变异的患者，TMB更有可能高：*RRM1*、*TP53*、*FANCE*、*NEIL1*、*POLE*、*POLG*、*FANCE*、*GEN1*、*RPA1*。而NSCLC中有确定药物治疗靶点突变的患者，如*EML4-ALK*融合、*EGFR*突变、*ROS1*重排、*BRAF*融合等，通常TMB表达较低。

一项研究纳入了147例肺癌患者，检测450个基因包括全部外显子及部分内含子。结果发现*KRAS*突变组的TMB值（中位TMB=10.6）明显高于野生型组患者（中位TMB=4.6，*P*=0.027）。*EGFR*突变组的TMB值明显低于*EGFR*野生型组（中位TMB=8.4，*P*=0.034）。TK融合组的TMB中位值为6.5，与其他组没有显著差异。15% *EGFR*野生型组患者的TMB值> 20，而只有6% *EGFR*突变组患者属于高TMB^[14]^。

鉴于全外显子测序（whole exome sequencing, WES）价格昂贵，Chen等^[15]^比较了目标基因组分析（targeted genomic profiling, TGP）和WES对27例NSCLC患者TMB的分析，发现TGP（811个基因）和WES结果具有高度一致性（R^2^ =0.71, *P* < 0.05）。同时发现*MMR*基因突变仅发生在高TMB的患者（错配修复基因为*MSH2*、*PMS2*）。对使用了Pembrolizumab治疗的34例患者进行评估，发现使用TGP及WES进行疗效预测的AUC分别为0.80、0.84。这些结果说明，TGP可以替代WES作为TMB检测技术。对8例患者的肿瘤组织及ctDNA同时检测TMB，发现结果也高度一致（*R^2^*=0.80, *P* < 0.05），说明可以考虑选择无创的液体活检替代有创的组织活检。

Iijima等^[16]^的研究也得到了相同的结论，14例接受nivolumab治疗的患者，在治疗前及治疗后的1周、2周、4周、6周、8周抽取外周血进行53个目标基因进行二代测序并与组织进行比较，结果发现高TMB患者用药2周内就可根据突变等位基因频率（mutation allelic frequency, MAF）下降情况判断疗效。

## 3 肿瘤驱动基因突变

除研究总的基因突变状况与免疫治疗有效性的关系外，人们也期望能发现某个驱动基因的突变能够预测免疫治疗的有效性。有研究^[17, 18]^表明*EGFR*突变或ALK重排的NSCLC患者使用PD-1/L1无效，机制尚不清楚。Liu等^[19]^的研究纳入745例患者，分为EGFR/ALK阳性组（344例）和EGFR并ALK阴性组（401例）。阳性组5.52% PD-L1^+^ /CD8^+^；11.92% PD-L1^-^ /CD8^+^；18.90% PD-L1^+^ /CD8^-^；63.66% PD-L1^-^ /CD8^-^。EGFR并ALK阴性组13.97% PD-L1^+^ /CD8^+^；6.98% PD-L1^-^ /CD8^+^；30.42% PD-L1^+^ /CD8^-^；48.63% PD-L1^-^ /CD8^-^。结果发现EGFR/ALK阳性患者PD-L1^+^ /CD8^+^对较低而PD-L1^-^ /CD8^-^则明显较高。EGFR/ALK阳性组患者的亚组5年生存率存在明显差异（PD-L1^+^ /CD8^+^ : 41.9%, PD-L1^-^ /CD8^+^ : 91.0%, PD-L1^+^ /CD8^-^ : 75.4%, PD-L1^-^ /CD8^-^ : 69.7%, *P*=0.003）。然而在EGFR并ALK阴性组差异则不显著（PD-L1^+^ /CD8^+^ : 66.5%, PD-L1^-^ /CD8^+^ : 76.9%, PD-L1^+^ /CD8^-^ : 62.3%, PD-L1^-^ /CD8^-^ : 70.6%, *P*=0.341）。但是使用Nivolumab治疗患者，虽然*EGFR*突变阳性患者疗效不佳，但是仍有部分患者明显获益，具体机制尚需进一步研究^[20]^。

## 4 免疫效应标志物

PD-1/PD-L1免疫治疗，是解除免疫抑制从而提高效应细胞杀伤的敏感性，激发和增强机体抗肿瘤免疫应答，以达到控制肿瘤的目的。所以，假设治疗有效，那么T细胞活化的下游一系列炎症因子理论上都应该升高，例如IFN-γ、TNF-α、IL-6等，所以，炎症因子可能是预测免疫治疗的标志物。

一项研究入组了17例NSCLC和21例黑色素瘤、接受nivolumab治疗的患者，发现INFγ与PFS、OS、疾病控制率（disease control rate, DCR）相关，INFγ中、高水平组PFS显著高于低表达组（5.1个月*vs* 2.0个月，*P*=0.012, 4），两组的OS虽然没有统计学差异，但中、高水平组要长于低水平组（10.2个月*vs* 4.9个月，*P*=0.068, 7）^[21]^。另有研究^[22]^表明，使用nivolumab或pembrolizumab的NSCLC患者，治疗1周内血清IL-6、CRP升高组有效率显著高于未升高组，而TNF-α升高组患者，未表现出显著的有效率。

Kowanetz认为效应T细胞（T-effector, Teff）的基因表达（gene expression, GE）情况可以反映免疫抑制剂治疗前的免疫状态。作者分析了753例使用atezolizumab的NSCLC患者的Ⅲ期临床试验（OAK）的结果。将*PD-L1*、*CXCL9*、*IFNγ*这3个基因作为Teff的标志，检测Teff GE，同时检测肿瘤组织PD-L1的表达情况。结果发现，Teff GE越高，PFS获益越显著。用Teff GE的中位值将患者分组，高表达组的PFS显著高于低表达组（HR=0.73, 95%CI: 0.58-0.91）。作者同时认为，使用Teff GE来预测atezolizumab的疗效较PD-L1更为敏感，因为目前数据显示使用Teff GE来预测，50%的患者可以有PFS的获益^[23]^。

## 5 血细胞计数

除以上指标外，血细胞计数等常规实验室指标在预测免疫治疗疗效方面也有较好效果。本次大会有多项研究对此进行了报道。

一项研究回顾性分析了134例晚期或者复发使用Nivolumab的NSCLC患者，分析患者实验室化验的常规指标，包括：中性粒细胞绝对计数（absolute neutrophil count, ANC）、淋巴细胞绝对计数（absolute lymphocytes count, ALC）、单核细胞绝对计数（absolute monocytes count, AMC）、嗜酸性粒细胞绝对计数（absolute eosinophil count, AEC）、CRP以及乳酸脱氢酶水平。多因素分析结果表明，低水平ANC、高水平ALC、高水平AEC是较好的PFS、OS的独立相关因素。将以上3个指标做不同分组分析，全部符合3个条件的患者预后最好，仅符合一个条件的，预后最差，中位PFS分别是209 d、87 d、42 d，药物有效率分别为43.5%、27.1%、5.9%^[8]^。另外一项研究，应用免疫抑制剂治疗2个周期后，ANC < 4, 000患者的OS明显延长（NR *vs* 4.9个月，*P*=0.02）。ALC升高患者的DCR有所增加（147 *vs* 155, *P*=0.05）。该研究还同时发现，肺腺癌患者肿瘤组织中TTF1表达水平与免疫治疗有效率相关（88% *vs* 45%, *P*=0.03），ALC≥1, 000患者的TTF1阳性率更高（82% *vs* 45%, *P*=0.05）^[24]^。一项应用nivolumab的研究发现，19例患者治疗前低水平ANC、中性粒细胞、淋巴细胞比值（neutrophil to lymphocyte ratio, NLR）和血小板、淋巴细胞比值（platelet lymphocyte ratio, PLR）生存期更长，治疗过程中NLR升高反映疾病进展^[25]^。

Soyano等^[26]^的研究纳入了157例应用nivolumab或pembrolizumab的NSCLC患者，结果表明治疗前高水平的ANC、AMC、NLR、MLR患者的预后更差。治疗前ANC/ALC≥5.9患者的死亡风险及疾病进展风险均显著高于ANC/ALC < 5.9患者（HR=1.65, 95%CI: 1.06-2.56, *P*=0.027）（HR=1.65, 95%CI: 1.17-2.34, *P*=0.005）。治疗前MLR≥11.3组患者死亡风险更高（HR=2.13, 95%CI: 1.32-3.44, *P*=0.002）。

## 6 综合分析模型

以上单一标志物的应用并不十分理想，目前有研究将涉及PD-1疗效的影响因素综合起来设计成数学模型来提高预测免疫治疗效果的准确性。

一项研究^[27]^将使用PD-1抗体的NSCLC患者提取361份关注区域（region of interest, ROIs）样本和254, 205个细胞核，总结出一个新型的数字病理系统，用以预测使用PD-1抗体的疗效。系统通过计数肿瘤的ROIs的细胞核的形态特征，包括大小、圆度、周长等与平均值或标准制定差异以及核内部特征（主要是染色质结构）就可以预测疗效。作者认为，细胞核的形态学特征要比多形性及异质性测量数据（pleomorphism and heterogeneity measurement data, CFLCM）还要重要。虽然本次分析的样本量不足够，结果稳定性可能受到影响，但是作者认为针对病理组织使用计算机系统进行预测疗效是可行的。

Wungki等^[13, 28]^则将性别（Sex）、ECOG评分、中性粒细胞与淋巴细胞比值（NLR）、治疗后NLR减治疗前NLR（Delta NLR=DNLR）等参数设计成iSEND计算模型，将使用nivolumab的患者进行验证，发现评分好的患者预后好，PFS分别为17.4个月*vs* 5.1个月（HR=0.32, 95%CI: 0.20-0.50, *P* < 0.000, 1）。

## 7 展望

免疫检查点抑制剂治疗肿瘤其效果除与肿瘤细胞免疫抑制信号的解除有关外，还与肿瘤免疫刺激信号强弱、免疫效应细胞功能的发挥及其他免疫抑制信号途径的活动有关，机制相对复杂，找到单一标志物有效筛选敏感患者的可能性较低。因此，该领域的研究重点一是寻找更适合的标志物，例如一项入组504例鳞癌及522例腺癌患者的研究发现，不论何种病理类型，EAM相关的基因过度表达均可以导致CD4/CD8 T细胞下降和B/Treg细胞增加，影响免疫治疗疗效^[29]^；二是利用大数据进行更深的挖掘，从而建立综合预测模型，多维度预测治疗敏感性。另外，对标志物检测技术和判别标准的统一化也很有必要。
